# Patterning with clocks and genetic cascades: Segmentation and regionalization of vertebrate versus insect body plans

**DOI:** 10.1371/journal.pgen.1009812

**Published:** 2021-10-14

**Authors:** Margarete Diaz-Cuadros, Olivier Pourquié, Ezzat El-Sherif

**Affiliations:** 1 Department of Genetics, Harvard Medical School, Boston, Massachusetts, United States of America; 2 Department of Pathology, Brigham and Women’s Hospital, Boston, Massachusetts, United States of America; 3 Harvard Stem Cell Institute, Harvard University, Cambridge, Massachusetts, United States of America; 4 Division of Developmental Biology, Department of Biology, Friedrich-Alexander-Universität Erlangen-Nürnberg, Erlangen, Germany; New York University, UNITED STATES

## Abstract

Oscillatory and sequential processes have been implicated in the spatial patterning of many embryonic tissues. For example, molecular clocks delimit segmental boundaries in vertebrates and insects and mediate lateral root formation in plants, whereas sequential gene activities are involved in the specification of regional identities of insect neuroblasts, vertebrate neural tube, vertebrate limb, and insect and vertebrate body axes. These processes take place in various tissues and organisms, and, hence, raise the question of what common themes and strategies they share. In this article, we review 2 processes that rely on the spatial regulation of periodic and sequential gene activities: segmentation and regionalization of the anterior–posterior (AP) axis of animal body plans. We study these processes in species that belong to 2 different phyla: vertebrates and insects. By contrasting 2 different processes (segmentation and regionalization) in species that belong to 2 distantly related phyla (arthropods and vertebrates), we elucidate the deep logic of patterning by oscillatory and sequential gene activities. Furthermore, in some of these organisms (e.g., the fruit fly *Drosophila*), a mode of AP patterning has evolved that seems not to overtly rely on oscillations or sequential gene activities, providing an opportunity to study the evolution of pattern formation mechanisms.

## Introduction

Segmentation, also known as metamerism, refers to the organization of the body into repeating units of similar structure along the anterior–posterior (AP) axis. Among bilaterian animals, several clades feature segmented body plans, including annelids, arthropods, tardigrades, cephalochordates, and vertebrates, among others [1]. Segmentation is usually coupled with regionalization, a process in which the animal body plan is divided into several fates, such that different segments along the AP axis of segmented organisms acquire different identities and develop distinct morphological features.

Insects could be classified into 2 broad categories: simultaneously segmenting (or long-germ) insects and sequentially segmenting (or short-germ) insects [2,3]. Recently, it has been shown that segmentation and regionalization in both vertebrates and short-germ insects share common mechanistic themes, a natural consequence of the fact that these processes rely on translating temporal gene and signaling pathway activities into spatial patterns [3–14]. Translating a temporal sequence into a spatial pattern seems to be a common strategy in development, as it has been discovered in other developmental processes such as lateral root formation in plants [15,16] and fate specification in insect neuroblasts [17–20], the vertebrate neural tube [21–23], and (arguably) the vertebrate limb bud [24–27]. This strategy is usually mediated by the interaction between a morphogen gradient and either a clock (to generate a periodic pattern) or a genetic cascade (to produce a nonperiodic pattern).

In this review, we use the processes of segmentation and regionalization in short-germ insects and vertebrates as model systems to investigate the basic principles of embryonic patterning via clocks and genetic cascades. We also investigate the evolution of such mechanisms into other modalities, such as those involved in the segmentation and regionalization in long-germ insects. We focus on the flour beetle *Tribolium castaneum* as a representative of short-germ insects and the fruit fly *Drosophila melanogaster* as a representative of long-germ insects, since they are the most studied species of their respective categories. We should bear in mind, however, that segmentation and regionalization in insects show remarkable evolutionary flexibility [28–32], and studying these processes in other insects, therefore, is necessary to gain a more comprehensive view of AP patterning in insects. We would like also to stress that our review is a study of the parallels between segmentation and regionalization in insects and vertebrates, rather than a comprehensive coverage of these processes. For more specialized in-detail coverage of these processes, we refer the reader to recent reviews such as [11,12,33–37].

In this review, we start by introducing an overview of segmentation and regionalization in insects and vertebrates and the associated morphogenetic movements and patterns of gene expression, contrasting along the way the analogous processes in the 2 groups of organisms. Then, we discuss in more detail the mechanistic underpinnings of these processes, their coupling, and their evolution. We find that vertebrates and short-germ insects share an impressive number of features related to segmentation and regionalization mechanisms, but also have important differences. We also find that segmentation and regionalization in long-germ and short-germ insects and vertebrates share interesting mechanistic similarities, suggesting deep similarities between pattern formation mechanisms that superficially seem radically different.

## An overview of segmentation and regionalization in insects and vertebrates

The main body axis of insects is organized into segments, and each group of segments (tagma) has specific identity: gnathal, thoracic, or abdominal (other segmented structures like antenna are not covered in this review). Segmentation of the insect AP axis is mediated by the periodic expression of a group of genes called “pair-rule genes,” whereas regionalization is mediated by the nonperiodic expression of a group of genes called “gap genes” (which also instructs/interacts with segmentation in some insects) [12,33]. Expressions of both pair-rule and gap genes are down-regulated shortly after they do their job, and segment boundaries and regional identities are further maintained by the expression of downstream segment polarity and Hox genes, respectively. The spatiotemporal dynamics of both pair-rule and gap genes along the AP axis differ among different insects, although the final expression patterns of downstream segment polarity and Hox genes are more or less conserved. For example, in a short-germ insect like *Tribolium*, both gap and pair-rule genes are expressed sequentially, whereas in a long-germ insect like *Drosophila*, both gap and pair-rule genes are expressed more or less simultaneously.

In vertebrate animals, 3 main distinct structures exhibit segmental organization along the AP axis: the somites that give rise to the axial skeleton, the rhombomeres of the hindbrain, and the pharyngeal arches [38]. Here, we focus on the segmentation of the musculoskeletal system as it encompasses the entire trunk and tail and thus represents the most significant form of segmentation in vertebrate animals. In adults, segmented tissues include the vertebral column and ribs, as well as the associated skeletal muscle, tendons, and ligaments [39]. Somitogenesis also provides the template for segmentation of other structures such as blood vessels and nerves. Segmentation is first established through the process of somite formation. Somites are bilaterally symmetric, epithelial blocks of paraxial mesoderm that bud off progressively from the anterior end of the presomitic mesoderm (PSM) [40]. Thus, unlike insects, segmentation always occurs in a sequential instead of simultaneous fashion in vertebrate organisms. The regionalization of segments is under the control of Hox genes and is most clearly exemplified by the distinct structure of vertebrae along the AP axis [41]. For instance, vertebrae at the thoracic level develop ribs, while those at the lumbar level do not. This morphological distinction is specified by Hox expression at the molecular level.

## Morphogenesis and gene expression patterns

### Axis elongation

**Fig 1:**
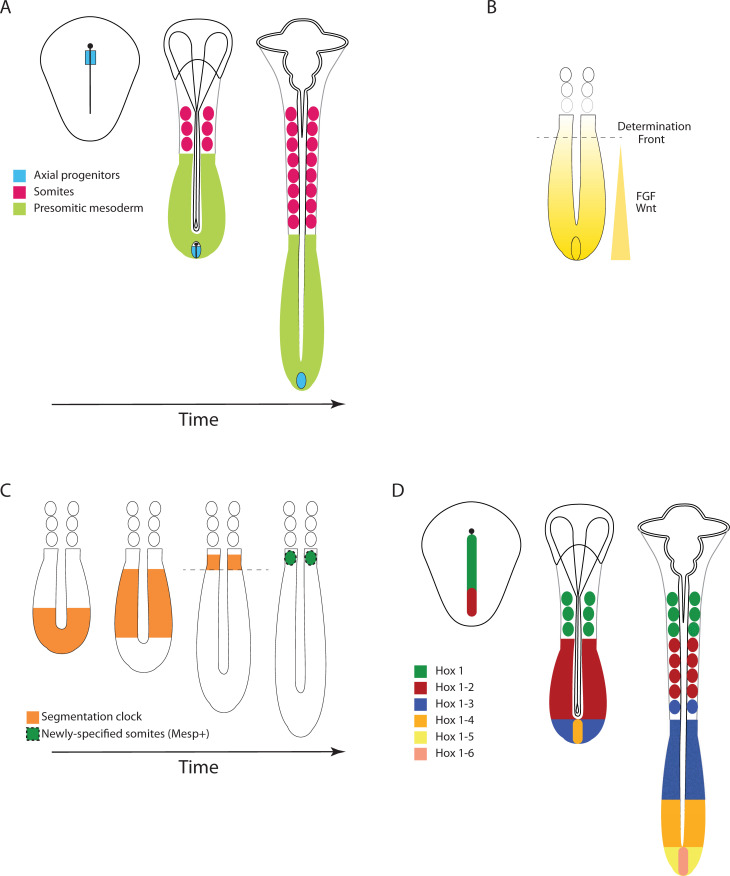
Gene expression patterns in vertebrates. **(A)** Morphogenesis in the chicken embryo. Left: Epiblast stage. The primitive streak is shown as a vertical line with the node on the top. Axial progenitors (blue) are located in the anterior region of the primitive streak. Middle panel: 3-somite stage. Paraxial mesoderm is segmented into somites (pink) anteriorly, but unsegmented posteriorly (PSM; green). Axial progenitors (blue) are located in the regressing primitive streak at the posterior end of the embryo, where axial elongation takes place. Right panel: 8-somite stage. Somites (pink) continue to from sequentially from the PSM (green), as the embryo elongates posteriorly. **(B)** Signaling gradients in somitogenesis. FGF and Wnt signaling (yellow) activity is highest in the posterior progenitor domain and forms a posterior-to-anterior gradient along the PSM. The determination front (dotted line) is positioned by these signaling gradients. **(C)** The segmentation clock. Waves of gene expression (orange) are initiated in the posterior domain and travel anteriorly along the PSM. When the segmentation clock reaches the determination front (dotted line), a new pair of somites is specified (green). **(D)** Hox gene spatial and temporal colinearity. The schematic depicts expression of a hypothetical Hox cluster in chicken embryos. Hox genes are first expressed in the progenitor domain and spread anteriorly through cell ingression, thus leading to the formation of nested expression domains (colored regions) through the sequential activation of more posterior Hox genes. Throughout the figure, for all embryo schematics: anterior to the top and posterior to the bottom. PSM, presomitic mesoderm.

Paraxial mesoderm is the primarily segmented tissue in vertebrate organisms. It flanks both sides of the axial structures (neural tube and notochord) in a bilaterally symmetrical manner. In somitogenesis stage embryos, the posterior part of the paraxial mesoderm, also known as the PSM, is unsegmented and includes highly motile mesenchymal cells [42] (Fig 1A). More anteriorly, the paraxial mesoderm is segmented into epithelial somites, which form rhythmically and sequentially in the anterior most region of the PSM (Fig 1A). As vertebrate embryos develop progressively from head to tail, somitogenesis takes place concomitantly with axis elongation. As somites bud off one after the other from the anterior end of the PSM, new mesodermal cells are continually generated from progenitors located in the posterior region of the embryo [43]. During anterior body formation (i.e., neck and trunk), new PSM cells arise by ingression in the anterior region of the regressing primitive streak or closing blastopore/shield. PSM cell fate specification is Wnt dependent, as the fate determinants T and Msgn1 are Wnt targets [44,45]. At these early stages, convergent cell movements toward the midline play an important role in axial elongation [46]. Similarly, axial stem cells located in the tail bud and known as the neuromesodermal progenitors (NMPs) are the source of new mesodermal tissue during posterior body formation (i.e., sacrum and tail) [43]. At these later stages, elongation is driven by a random motility gradient in the posterior PSM [42]. Importantly, however, cell division is not limited to the progenitor domain nor oriented along a particular axis [47].

**Fig 2:**
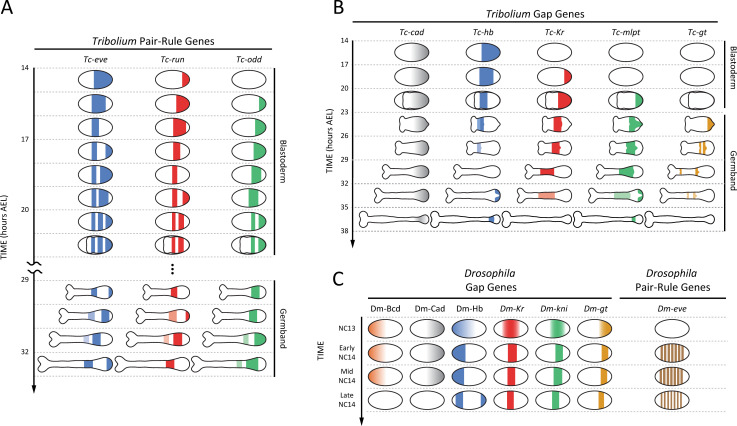
Key gene expression patterns during segmentation and regionalization in *Tribolium* (as a representative of short-germ insects) and *Drosophila* (as a representative of long-germ insects). **(A)** Expression patterns of segmentation clock genes in *Tribolium* (namely primary pair-rule genes: *Tc-eve* (shown in blue), *Tc-run* (red), and *Tc-odd* (green)) across time (at 24°C) during both the blastoderm and germ-band stages of embryogenesis. *Tribolium* clock genes are expressed periodically as consecutive waves that propagate from posterior (right) to anterior (left) (frequency doubling of pair-rule gene expressions is not depicted). **(B)** Key regionalization gene expression patterns in *Tribolium* (namely gap genes: *Tc-hb* (blue), *Tc-Kr* (red), *Tc-mlpt* (green), and *Tc-gt* (gold)) across time (at 24°C) during both blastoderm and germ-band stages of embryogenesis (head expressions of gap genes are not depicted for simplicity), in addition to *Tc-cad* (shown in gray), a master regulator of both segmentation and regionalization in *Tribolium*. *Tribolium* gap genes are expressed as consecutive nonperiodic waves that propagate from posterior (right) to anterior (left) within the expression domain of *Tc-cad*. **(C)** Expression patterns of selected *Drosophila* segmentation genes (namely the pair-rule gene *Dm-eve* (shown in brown)) and regionalization genes (namely the gap genes: Dm-Hb (blue), *Dm-Kr* (red), *Dm-kni* (green), and *Dm-gt* (gold)) across time during the blastoderm stage, in addition to master regulator gradients: Dm-Bcd (orange) and Dm-Cad (gray). *Drosophila* gap gene expression bands arise more or less simultaneously before nuclear cycle 14 (NC14). Later during NC14, pair-rule gene expressions arise, also more or less simultaneously. Eventually, expression domains of both gap and pair-rule genes undergo posterior-to-anterior shifts, reminiscent of the posterior-to-anterior propagation of gap and pair-rule gene expression waves in *Tribolium*. Finally, and concomitantly with the degradation of Dm-Bcd and Dm-Cad gradients, gap and pair-rule gene expression domains stabilize, pair-rule gene expressions undergo frequency doubling (not depicted), then both gap and pair-rule gene expressions eventually fade. Throughout the figure, for all embryo schematics: anterior to the left and posterior to the right.

In insects, anterior segments form in the “blastoderm,” a single layer of nuclei (that eventually cellularize) covering the entire egg cortex. The embryo then enters the “germ-band” stage where the AP axis elongates, and concomitantly, posterior segments form sequentially from a segment addition zone (SAZ) [2] (Fig 2A). The number of segments that form in the blastoderm versus those that form in the germ-band is different in different species. In the so-called “short-germ” insects, most segments form concomitantly with axis elongation during the germ-band stage. In “long-germ” insects, most segments form during the blastoderm stage before the transition into the germ-band stage where the whole (already segmented) embryo undergoes axis elongation [2]. Many insects fall somewhere between these 2 extreme cases, where short-germ embryogenesis is thought to be the ancestral mode of embryogenesis in insects and arthropods in general [12,48]. AP patterning in short-germ insects is usually (but not always) sequential [3,6,7,49], whereas AP patterning in long-germ insects is usually (but not always) simultaneous (with an occasional hint of sequentially [9,50–53]). Therefore, the terms “short-germ insects” and “long-germ insects” are sometimes used synonymously with “sequentially segmenting insects” and “simultaneously segmenting insects,” respectively. Axis elongation of insect germ-bands is driven by convergent extension and/or cell proliferation [7,54–59].

Segment formation in the insect blastoderm, when the embryo is not elongating, might be compared with the formation of prechordal and head mesoderm in vertebrates. In chicken embryos, these tissues are patterned at the epiblast stage before axis elongation ensues. Thus, in both insects and vertebrates, patterning of the most anterior parts of the body tends to differ from the rest of the axis.

### Segmentation clock in vertebrates and short-germ insects

Segmentation of the AP axis in vertebrates and short-germ insects is achieved rhythmically and sequentially through the oscillatory activity of a set of genes whose expression transverses the AP axis from posterior to anterior, thus generating wave-like patterns (Figs 1C and 2A). Each cycle of oscillatory gene expression triggers the specification of a single segment (in vertebrates and some arthropods) or a pair of segments (in most insects).

In short-germ insects, most of segmentation genes with oscillatory expression belong to a subset of a group of transcription factors (of different molecular classes) called “pair-rule genes” (e.g., *even-skipped* (*eve*), *runt* (*run*), and *odd-skipped* (*odd*) in the flour beetle *Tribolium*) [6,7,60] (Fig 2A). Other “secondary” pair-rule genes (e.g., *paired* (*prd*) and sloppy-paired (*slp*)) do not exhibit oscillatory expressions and are rather involved in later phases of segmentation [60]. Components of the Notch signaling pathway and their target *hairy* (which is a pair-rule gene in many arthropods) were also found to oscillate in some arthropods (e.g., cockroach [61], spider [62,63], centipede [64,65], and a crustacean [66]) but not in others (e.g., *Tribolium*, where *notch* and *delta* are not expressed periodically, while *hairy*, although expressed periodically, is, arguably, not involved in trunk segmentation [12,60,67–69]). In vertebrates, *hairy*/*enhancer of split* (*Hes/Her*) transcription factors and components of one or several signaling pathways have been shown to oscillate (e.g., Notch in zebrafish and Notch, Wnt, and FGF in amniotes) [70]. Core components of the Hippo signaling pathway (*Tead4*, *Amotl2*, and *Cyr61*) also appear to oscillate in mouse and human PSM [71,72]. However, not all vertebrate cyclic genes are related to signal transduction pathways, as some Hox genes, histone modifiers, and metabolic enzymes have also been detected to oscillate in amniotes [70]. Overall, the amniote segmentation clock encompasses a much larger and more complex network of oscillating genes compared to lower vertebrates and insects (although probably not all components of insect segmentation clock have been discovered yet).

Within a given short-germ insect or vertebrate species, all cyclic genes share the same oscillatory period (which might vary along the SAZ and PSM) but not the same oscillatory phase. In the short-germ insect *Tribolium*, each pulse of the *eve* expression wave is chased in space and time by a pulse of *runt*, which, in turn, is chased by a pulse of *odd* [7,60] (Fig 2A). In amniotes, Wnt targets oscillate in opposite phase to Notch and FGF pathway components in the posterior PSM [70,73–75]. These phase relationships change in the anterior PSM and are thought to potentially encode patterning information [76].

### Morphogen gradients and segmental readouts in vertebrates and short-germ insects

Segmentation clock genes are expressed in dynamic waves with fast dynamics at the posterior end of the PSM in vertebrates and SAZ in short-germ insects. However, they gradually slow as they exit the posterior PSM and SAZ to form transiently stable gene expression domains. The transiently stable segmentation gene expressions just anterior to the PSM and SAZ regulate the expression of boundary markers, such as *Mesp* and *Ripply* in vertebrates [77,78] and segment polarity genes (e.g., wingless and hedgehog) in insects [79–81]. The posterior PSM and SAZ are believed to possess certain qualities that keep cells undifferentiated and their anterior border is thus known as a “determination front,” since cells passing through it assume their segmental fate [82]. In both vertebrates and arthropods, genes involved in setting up segment boundaries (*Mesp* and segment polarity genes, respectively) are also responsible for patterning the AP polarity of segments by specifying rostral/caudal compartments [83,84].

An important difference between vertebrates and short-germ insects is that while each tick of the segmentation clock specifies 1 segment in vertebrates, it specifies 2 segments in short-germ insects. Upon crossing the determination front, however, the expression of primary pair-rule genes undergoes frequency doubling and regulate the late expressions of secondary pair-rule genes [6,13,49,60]. The now segmental expression of both primary and secondary pair-rule genes specify the segmental expressions of segment polarity genes [80].

In both short-germ insects and vertebrates, the determination front is (possibly indirectly) positioned by the posterior-to-anterior signaling gradients of Wnt (e.g., Wnt3a in vertebrates and Wnt1 and Wnt8 in *Tribolium*), which activate a downstream gradient of *caudal* (*cad*/*cdx*) [3,8,73,85–89] (Figs 1B and 2B). In vertebrates, but in none of the insects examined so far [90], a parallel gradient of FGF (e.g., Fgf8 and Fgf4) forms a positive feedback loop with Wnt in the PSM and is similarly crucial to specify the determination front [91]. These signaling molecules are most highly expressed in the posterior progenitor domain, resulting in a continuous regression of the determination front coordinately with the elongation of the vertebrate PSM and germ-band of short-germ insects [3,13,92,93]. During the blastoderm stage of insects, however, the Wnt/Cad gradient exhibits a buildup followed by decaying dynamics but does not retract until the onset of gastrulation [8]. Whereas classical models conceptualized the wavefront as a simple threshold of signaling activity, more recent work suggests that cells actually read out the spatial fold change in FGF signaling rather than the absolute ligand concentration [94].

In vertebrates, a counter gradient of retinoic acid (RA), which is secreted by the somites and anterior PSM, antagonizes the FGF gradient and contributes indirectly to positioning the determination front at early stages [95,96]. However, this RA counter gradient is expendable for wavefront positioning after E9.5 in mouse embryos as well as in zebrafish embryos [94,97]. Counter gradients of *axin* (*axn*) [85], zerknüllt (*zen*) [98], *orthodenticle* (*otd*) [99], and Hunchback (Hb) [100] exist only in the initial phase of segmentation (the blastoderm stage) in *Tribolium*. Recently, the expression of *Dichaete* and the pioneer factor *odd-paired* (*opa*) where reported to form staggered wavefronts along with the Wnt/Cad gradient throughout segmentation in *Tribolium* [13].

### Regionalization in vertebrates and short-germ insects

Across bilateria, in both segmented and unsegmented animals, Hox genes are responsible for dividing the AP axis into different fates (e.g., thoracic and abdominal fates), a process usually termed “regionalization.” In segmented animals, Hox genes are expressed in nested domains along the AP axis (Fig 1D) and specify segmental identity. Even though the number and complexity of Hox clusters varies significantly across phyla, the patterning role of orthologous Hox genes is subject to deep evolutionary conservation. For instance, in all the model organisms described in this review (*Drosophila*, *Tribolium*, zebrafish, chicken, and mice), orthologs of *AbdB* specify the identity of the most posterior body segments [101]. A central property of Hox genes is their spatial colinearity: In animals with intact Hox clusters (like all vertebrates and insects such as *Tribolium*), the 3′ to 5′ arrangement of Hox genes along the chromosome matches the order of their expression along the AP body axis [102,103]. In animals with a partially fragmented Hox cluster (like *Drosophila*), it is common that the principle of colinearity is preserved within each intact subcluster. In animals whose AP axis is segmented sequentially (e.g., vertebrates and short-germ insects), Hox genes also appear sequentially in time in an order that matches their arrangement along the chromosome, a phenomenon called “temporal colinearity” [104–107]. However, no temporal colinearity was observed in animals with a simultaneous mode of segmentation like *Drosophila*.

Whereas Hox genes are the final determinant of axial identity in all animals, they do not always play a specifying role. In insects, the initial driver of AP regionalization is a group of genes called “gap genes” (e.g., *hb*, *Krüppel* (*Kr*), and *giant* (*gt*)), whose expression precedes and regulates those of Hox genes [33,108–114]. In vertebrates and noninsect arthropods, a gene category corresponding to insect gap genes, to our knowledge, has not been identified [115], and Hox genes are likely to play both specification and determination roles. In the short-germ insect *Tribolium*, gap genes are expressed in sequential nonperiodic waves that propagate from posterior to anterior during both the blastoderm and germ-band stages [3] (Fig 2B). Although Hox genes are expressed sequentially in *Tribolium*, they exhibit no (or limited) wave dynamics and arise later in time within the anterior margin of the SAZ, suggesting that Hox genes act as readouts of gap genes in this insect.

An important point of comparison between AP patterning in vertebrates and short-germ insects is the timing of regionalization compared to segmentation. In all segmented animals, segmentation and regionalization have certain species-specific registry that grant each segment the appropriate AP fate. In *Tribolium*, gap gene sequential activation runs in parallel to the segmentation clock so that the temporal registry of gap and pair-rule genes matches their spatial registry along the AP axis [3,6,33,113] (compare the timing in Fig 2A and 2B). In amniotes, on the other hand, Hox genes are sequentially activated before (and in fact regulate) cell ingression into the PSM, and therefore, precede the corresponding ticks of segmentation clock [116,117].

### Segmentation, regionalization, and morphogen gradients in long-germ insects

The final expression patterns of segmentation genes (i.e., pair-rule genes) in both short-germ and long-germ insects are quite similar and are in the form of periodic stripes [49]. The final expression patterns of regionalization genes (i.e., gap genes) in both short-germ and long-germ insects are similar as well and are in the form of nonperiodic gene expression bands [113]. However, the spatiotemporal dynamics leading up to these final gene expression patterns are different in the 2 groups of insects. In the long-germ insect *Drosophila*, unlike *Tribolium*, the early determinants of regionalization (namely gap genes) are expressed earlier than (and regulate) segmentation, and both gap and pair-rule gene expressions arise more or less simultaneously during the blastoderm stage [29,33,34,118,119] (Fig 2C; compare with Fig 2A and 2B).

Four maternal gradients exist in the *Drosophila* blastoderm that have been implicated in AP patterning [33,120]: (i) the anterior-to-posterior gradient of Bcd [121–123]; (ii) the anterior-to-posterior gradient of maternal Hb [124–126]; (iii) the posterior-to-anterior gradient of Cad [127,128]; and (iv) the posterior-to-anterior gradient of Nanos (Nos) [33] (shown for Bcd, Cad, and Hb in Fig 2C). At the end of the blastoderm stage, and during gastrulation, all AP gradients (shown Bcd and Cad gradients in Fig 2C) decay along with gap gene expression domains [129], and concomitantly, the expressions of some of pair-rule genes undergo frequency doubling and segment polarity, and Hox gene expressions arise simultaneously (with a slight posterior to anterior progression).

## Mechanisms of segmentation and regionalization in insects and vertebrates

In the previous section, we described the spatiotemporal dynamics of gene expression and signaling pathway activities during regionalization and segmentation of the AP axis in insects and vertebrates. Here, we describe the mechanisms that mediate these expression patterns.

### Mechanisms of regionalization and segmentation in the long-germ insect *Drosophila*

#### Regionalization in *Drosophila*: Gap and Hox gene regulation

**Fig 3:**
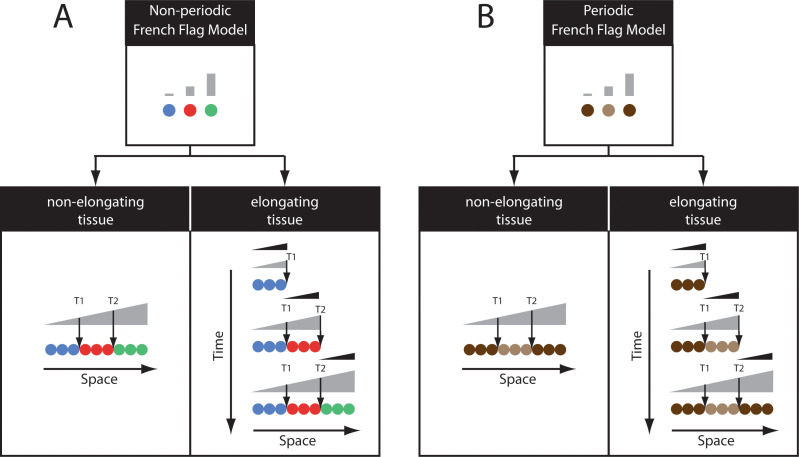
The FF model (see also Box 1). **(A)** Top panel: In the FF model, different concentrations of a morphogen gradient (shown in gray) activate different cellular states (shown in different colors). The FF model can pattern nonelongating tissues (bottom left) via a nonregressing gradient (shown in gray), as well as elongating tissues (bottom right) if a retracting short-range gradient (shown in black) activate a slowly decaying morphogen (gray). **(B)** In a similar fashion, the FF model can generate periodic patterns if a complex regulatory logic of periodically expressed genes (shown in brown) is employed or if an intermediate step of nonperiodic patterning is introduced. FF, French Flag.

**Fig 4:**
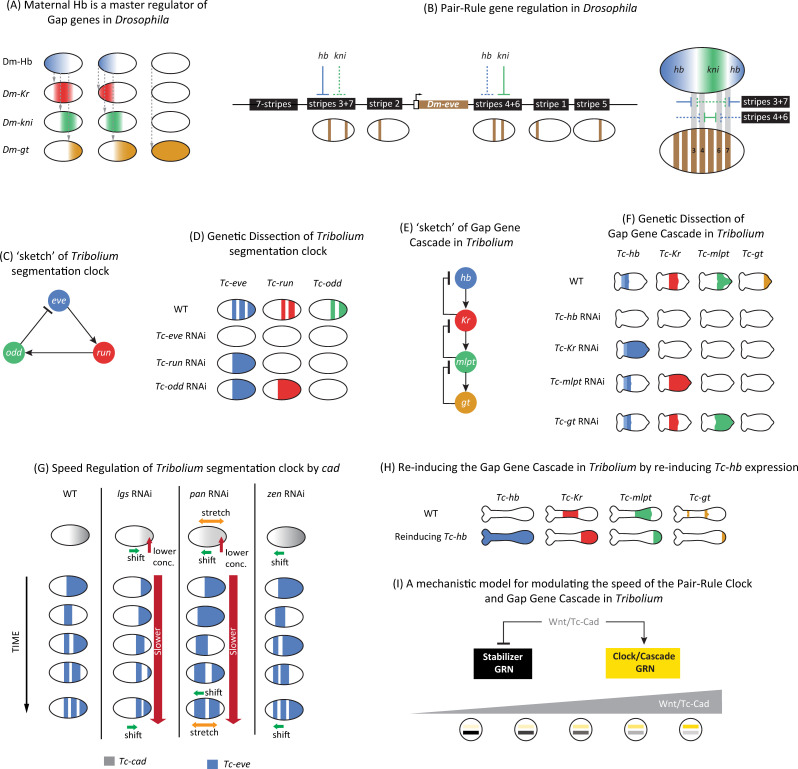
Segmentation and regionalization mechanisms in insects. **(A)** Maternal Dm-Hb gradient acts as a master regulator of gap genes in *Drosophila*. Progressive reduction of maternal Dm-Hb gradient (in various mutant backgrounds) results in progressive shifts of gap gene domains toward anterior. **(B)** Pair-rule stripes (shown for *Dm-eve*) are specified in a stripe-specific fashion in *Drosophila*. Left: Each one or pair of *Dm-eve* stripes are specified by a specific enhancer that receives inputs from upstream gap genes (shown repressive gap inputs for the 3+7 and 4+6 enhancers; strong repression shown in sold lines; weak repression in dashed lines). The full 7-stripe pattern is then stabilized by a 7-stripe (or zebra) enhancer. Right: Shown how the regulatory logics of 3+7 and 4+6 *Dm-eve* enhancers translates upstream gap gene expressions (shown are those of *Dm-Kr* and *Dm-kni*) into stripe pairs [140]. **(C)** A sketch of the genetic wiring of the *Tribolium* segmentation clock, composed of the 3 primary pair-rule genes: *Tc-eve*, *Tc-run*, and *Tc-odd*, wired into a negative feedback loop. Note that this is a parsimonious wiring explaining observed gene expression dynamics in WT and knockdown phenotypes. Actual wiring might differ from the one shown, especially that pair-rule genes are known to act as repressors rather than activators. **(D)** Experimental evidence of segmentation clock wiring in *Tribolium*. **(E)** A sketch of the genetic wiring of the *Tribolium* gap gene cascade. Note that this is a parsimonious wiring explaining observed gene expression dynamics in WT and knockdown phenotypes. Actual wiring might differ from the one shown, especially that most gap genes are known to act as repressors rather than activators. **(F)** Experimental evidence that gap genes are wired into a genetic cascade in *Tribolium*: repressing a single gap gene results in the up-regulation of upstream genes in the cascade and down-regulation of downstream genes. **(G)** Experimental evidence that the Wnt/Cad gradient (shown in gray) acts as a speed regulator of the segmentation clock in *Tribolium*. In *Tc-lgs* RNAi embryos: *Tc-cad* gradient is reduced (i.e., its peak concentration is lower than in WT) and shifted toward posterior; concomitantly, *Tc-eve* oscillation frequency is reduced and *Tc-eve* waves are shifted toward posterior. In *Tc-pan* RNAi embryos: *Tc-cad* gradient is reduced, shifted toward anterior, and stretched; concomitantly, *Tc-eve* oscillation frequency is reduced and *Tc-eve* waves are shifted toward anterior and stretched. In *Tc-zen* RNAi embryos: *Tc-cad* gradient has the same peak level and slope as in WT, but just shifted toward anterior; concomitantly, *Tc-eve* waves are shifted toward anterior without any sign of spatial stretch or time dilation. **(H)** Further evidence that gap genes are wired into a genetic cascade in *Tribolium*. Upon reinducing the leading gap gene in the cascade (*Tc-hb*) using a transgenic line where *Tc-hb* minigene is placed downstream of a heat-shock promoter, the whole gap gene sequence is reinduced in the SAZ. **(I)** A possible model for how the speed of the pair-rule clock or the gap gene cascade is modulated by a Wnt/Cad gradient in *Tribolium*: Wnt/Cad activates the pair-rule clock and/or gap gene cascade, but represses a multistable gene regulatory network. The gradual switch between the 2 gene networks results in the gradual slowing down of pair-rule oscillations and/or gap gene sequential activation. Throughout the figure, for all embryo schematics: anterior to the left and posterior to the right. RNAi, RNA interference; WT, wild-type.

The French Flag model, in which different concentrations of a morphogen gradient turn on or off different genes (Box 1; Fig 3) [130], has been the prime theoretical framework for how gap gene expressions are initialized in the early *Drosophila* embryo. Modulating the maternal expression of Hb alters the positioning of gap gene expression domains, in a fashion consistent with a French Flag model in which maternal Hb acts as a master morphogen gradient (Fig 4A) [125,126,131]. Later in time, cross-regulatory interactions between gap genes themselves refine their final expression patterns [34]. However, recent data, while confirming the primary role of the Hb gradient in regulating gap gene expression, indicate that gap gene regulation is much more dynamic [50,52,132] and follows a temporal mode of patterning similar to that observed in short-germ insects [9,51]. We discuss this point in more detail in the section “Toward reconciliation of simultaneous and sequential models of patterning.”

**Box 1. The French Flag model**

The French Flag model is one of the earliest models of pattern formation in development [130]. In this model, a morphogen gradient spans a tissue, and different morphogen concentrations specify different fates along the gradient (represented by different colors in Fig 3A (top)), where each fate is typically specified by the activity of 1 or more genes. Consequently, borders between consecutive gene expression domains along the tissue are specified by a series of thresholds of the morphogen gradient (e.g., T1 and T2 in Fig 3A, bottom left).

In the earliest formulation of the French Flag model, the morphogen gradient was assumed to be static (i.e., does not change in shape or concentration in space and time), suitable for partitioning a nonelongating tissue (Fig 3A, bottom left). In this scheme, long-range morphogen gradients are required to pattern large tissues. This is, however, hard to achieve in cases where tissue sizes are much larger than the range of influence of typical signaling pathways. Large tissues, in fact, are usually patterned during tissue growth. In such cases, a signaling center tethered at the elongating end of the tissue results in a regressing short-range gradient.

The French Flag model, however, can be modified to mediate patterning using a regressing short-range gradient (Fig 3A, right) [135]. In this scheme, a regressing short-range gradient (shown in black in Fig 3A, right) activates a gene whose product is of negligible decay rate (shown in gray in Fig 3A, right). In this way, a long-range gradient will span the full length of the tissue by the end of the tissue elongation phase. Such long-range gradient can then mediate patterning via a typical French Flag mechanism (Fig 3A, right).

We note here that the French Flag model can also mediate periodic patterning, if a patterning gene (like the one shown in brown in Fig 3B) is repeatedly activated then inactivated by a continuous range of morphogen gradient concentrations (Fig 3B). This scheme, however, requires patterning genes to be under the control of complex *cis*-regulatory logics. Alternatively, such a scheme can be mediated by an intermediate stage of nonperiodic patterning, a mechanism implicated in regulating pair-rule genes in *Drosophila*.

Approaching the end of the blastoderm stage, gap genes initialize downstream Hox genes [108]. Hox gene expressions are then refined by cross-regulatory interactions among Hox genes themselves [133] and modulated by the periodic expression of pair-rule genes [109,134].

#### Segmentation in *Drosophila*: Pair-rule and segment polarity gene regulation

**Fig 5:**
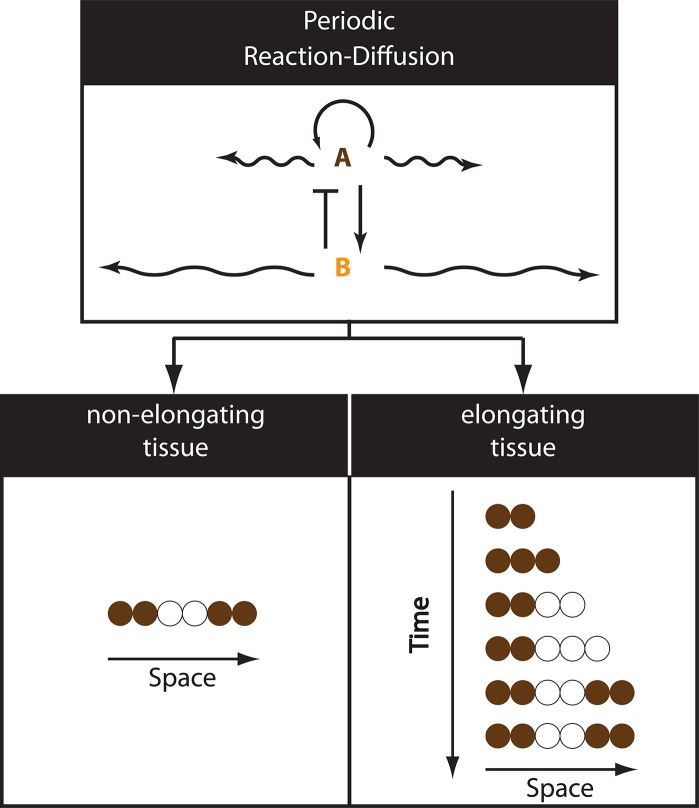
The RD model (see also Box 2). In the RD model (top panel), a slow-diffusing molecule (molecule A) activates itself as well as a fast-diffusing molecule (molecule B), which is also an inhibitor of A. The RD model can pattern nonelongating (bottom left) as well as elongating tissues (bottom right). RD, reaction–diffusion.

The simple periodic pattern of pair-rule genes tempted computational modelers to postulate that they are elegantly regulated by a reaction–diffusion mechanism (Box 2; Fig 5; [136,137]). However, experimental evidence showed that pair-rule genes are mainly regulated (rather inelegantly, [138]) by another round of the French Flag model. In this scheme, each pair-rule stripe (or pair of stripes) is mediated by a separate “stripe-specific enhancer.” Each stripe-specific enhancer is regulated in a dose-dependent manner by the upstream gradients of gap genes and various maternal factors (e.g., Bcd and Cad), whose identities are different depending on the specific location of the stripe along the AP axis of the embryo [139,140] (Fig 4B) (see [12,119] for more detailed discussion of pair-rule gene regulation in *Drosophila*).

**Box 2. The reaction–diffusion model**

Reaction–diffusion is a pattern formation mechanism in which a set of diffusible molecules interact and spontaneously pattern an initially homogeneous tissue [148–151]. The simplest and most popular instance of the reaction–diffusion model is the “local autoactivation and lateral inhibition” mechanism [147,152], where a slowly diffusing molecule (say, A; see Fig 5) can activate itself as well as a fast-diffusing molecule (say, B). Molecule B in its turn can inhibit A’s activity. Due to the ability of molecule A to self-activate, random fluctuations in A’s activity are occasionally amplified, forming localized domains of high A concentration in space, activating in their turn, corresponding domains of B activity. Due to the high diffusivity of molecule B, its activity domains then inhibit nearby A activity domains. This eventually leads to the formation of periodic patterns of A and B concentrations (Fig 5). The shapes of these activity domains (whether dotted or striped) and their characteristics (e.g., spacing between activity domains) are determined by system parameters and boundary conditions. The “local autoactivation and lateral inhibition” model, however, is one instance of the reaction–diffusion model, and it has been shown recently that the condition of differential diffusivities of molecules A and B can be relaxed if systems with more than 2 components are utilized [149,153].

Reaction–diffusion models have been implicated in the formation of various patterns during development, most of which are periodic [154,155]. The formation of nonperiodic patterns within the reaction–diffusion framework would require the employment of large number of diffusing molecules and/or signaling pathways, a condition that is hard to realize in most embryonic tissues.

After this initial phase of stripe specifications, the full expression pattern of pair-rule genes is then stabilized and/or refined and goes into a phase of frequency doubling. These late effects seem to depend on a rewiring of the pair-rule network, mediated by late-acting enhancers. Each of these late-acting enhancers drives the full pair-rule pattern (7 stripes in *Drosophila*) in enhancer reporter assays, and, hence, are sometimes called “7-stripe” or “zebra” elements [141] (Fig 4B). This rewiring of the pair-rule network (and possibly other genes involved in AP and dorsoventral patterning in the early *Drosophila* embryo) seems to be mediated by timing factors encoded by 2 pioneer factors: Zelda and Opa, where Zelda is responsible for activating the early network and Opa for the late network [142–144]. Indeed, in *opa* mutants, the frequency doubling of pair-rule genes is lost in *Drosophila* [142]. Dichaete was also recently suggested to mediate the transition from early to late patterning [13]. Interestingly, *cad* (potentially along with *zelda*), *dichaete*, and *opa* were found to be activated sequentially in the *Drosophila* embryo, reflecting a similar sequential activation in space and time in the *Tribolium* embryo [13,145].

As gap gene expressions provide positional information for downstream pair-rule genes, pair-rule gene expressions, likewise, have been suggested to provide positional information for downstream segment polarity genes in a yet another round of the French Flag model [146]. However, it was recently suggested that pair-rule gene expression concentrations are not read out in an ON–OFF fashion, but rather the temporal sequence of some of pair-rule gene expressions (mediated by the posterior-to-anterior shifts of gap genes, discussed in section “Toward reconciliation of simultaneous and sequential models of patterning”) encode the positional information for other pair-rule genes as well as segment polarity genes [14].

Finally, the reaction–diffusion model (Box 2; Fig 5), although had long been denied to play a role in *Drosohpila* segmentation, was later suggested to stabilize and maintain the final expression patterns of segment polarity genes [147].

### Mechanisms of segmentation in vertebrates and short-germ insects

**Fig 6:**
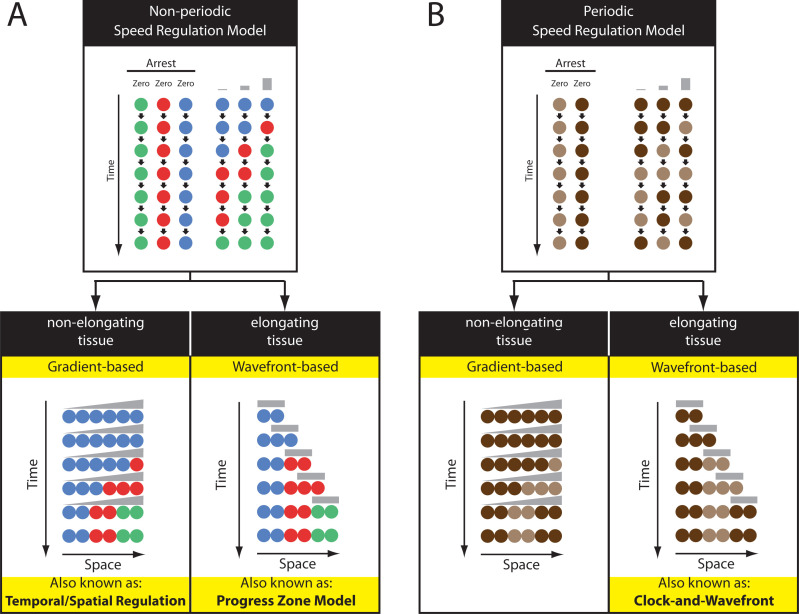
The SR model (see also Box 3). **(A) Top panel:** In nonperiodic SR model, a speed regulator (shown in gray) modulates the speed of cellular state transitions (each cellular state is shown in a different color) in a dose-dependent fashion (top panel, right). At a very low or zero concentration of the speed regulator, cellular state transitions are arrested (top panel, left). **(A) Bottom panel:** The SR model can operate in a gradient-based mode to pattern nonelongating tissues (left) or in a wavefront-based mode to pattern elongating tissues (right). **(B)** If the processes driving cellular state transitions is periodic (i.e., driven by a clock, which expression is shown in brown; top panel), the SR model can generate periodic patterns in both elongating and nonelongating tissues (via a wavefront-based and gradient-based modes of the model, respectively). SR, speed regulation.

The predominant view is that segmentation takes place in vertebrates and short-germ insects through a clock and wavefront mechanism (Box 3; Fig 6B, bottom right panel). This model was first proposed by Cooke and Zeeman in 1976 to be the underlying mechanism of vertebrate segmentation [156]. The basis of this model as originally formulated was that a catastrophe leading to abrupt changes in cellular properties takes place in the anterior PSM and underlies somite formation. The periodicity of this catastrophe is controlled by an oscillator that interacts with a slowly regressing maturation front, also known as the wavefront [156]. When a specific phase of the oscillator hits the wavefront, the catastrophe is triggered and results in somite individualization. Experimental evidence for the clock and wavefront model was not available until more than 20 years after its initial publication, when oscillations in the expression of the transcription factor *cHairy1* were discovered in the chicken PSM [4], and later on, oscillations in the expression of pair-rule genes *eve* and *odd* were discovered in the *Tribolium* SAZ [6,7]. Since then, molecular characterization of the clock and wavefront components has lent credibility to the model.

**Box 3. The speed regulation model**

**Model**

The speed regulation model is a synthesis of various patterning schemes that all rely on a single core mechanism [3,174]: the ability of a morphogen gradient to modulate the speed of a temporal sequence, either periodic or nonperiodic. In the nonperiodic version of the speed regulation model, each cell within a tissue has the capacity to transit through successive states (shown in different colors in Fig 6A, top; each state is defined by the expression of 1 or several genes), where the *speed* of state transitions is regulated by a molecular factor (shown in gray at the top of Fig 6A, and henceforth called a “speed regulator”). At very low or zero concentration of the speed regulator, state transitions become so slow that states are indefinitely stabilized (Fig 6A, top left). If a group of cells is subject to a gradient of the speed regulator (Fig 6A, bottom left), all cells go through successive states, but with slower and slower speed as we go from higher to lower values of the speed gradient. This gives the appearance that cellular states propagate as waves in the high-to-low direction of the gradient. Note that such waves do not require diffusion or cell–cell communication and, hence, are called “kinematic” or “pseudo-waves” [175–180]. We call this version of the model “gradient-based speed regulation,” which is well suited for patterning nonelongating tissues (Fig 6A, bottom left). The speed regulation model can also pattern elongating tissues if the gradient is retracting as a wavefront (henceforth called “wavefront-based speed regulation” model; Fig 6A, bottom right). In the same fashion, the speed regulation can partition a tissue into periodic structures if the sequential gene activation process is simply replaced with a clock (Fig 6B). Note that in the wavefront-based version of the speed regulation, if the wavefront is in the form of a tapered gradient (which can be seen as superposition of the gradient-based and the wavefront-based modes of the speed regulation model), kinematic waves will propagate from high to low concentrations of the gradient (opposite to the direction of wavefront retraction) as the tissue is elongating.

**The speed regulation model in development**

Special cases of the speed regulation model have been previously implicated in patterning various embryonic tissues. A nonperiodic version of the wavefront-based speed regulation model (Fig 6A, bottom right) was termed the “progress zone” model [27,181] and was (arguably [182,183]) suggested to pattern the vertebrate limb bud and was again termed the “temporal regulation” model and implicated in *Drosophila* neurogenesis [19,20].

The clock and wavefront model [156], originally suggested as an underlying mechanism of vertebrate somitogenesis, is, in fact, a periodic version of the wavefront-based speed regulation model (Fig 6B, bottom right). Careful inspection of the axis elongation phases of vertebrate and insect segmentation indicated that segmentation genes in these species are expressed in waves that propagate opposite to the direction of wavefront retraction [4]. This observation was reconciled with the clock and wavefront model by assuming that the wavefront is mediated by a tapered concentration gradient that modulates the frequency of clock oscillations (a model developed by Julian Lewis in [4]), which is a superposition of the gradient-based and wavefront-based modes of a periodic speed regulation model (Fig 6B). Indeed, FGF signaling was to found to fit the proposed criteria for a speed regulator, as its activity level was found to modulate the speed of the segmentation clock in an in vitro assay [184]. Similar oscillatory waves were observed during the blastoderm stage of *Tribolium* segmentation [6]. These waves were hypothesized to be mediated by a static frequency gradient, which is a periodic version of a gradient-based speed regulation model (Fig 6B, bottom left) [6]. A similar model was used to explain the oscillatory waves observed in PSM ex vivo cultures [185]. Nonperiodic versions of these models were then suggested to mediate gap gene regulation in *Tribolium* [3]. Finally, a nonperiodic version of the gradient-based speed regulation model (Fig 6A, left) was coined the “temporal/spatial patterning” model and was implicated in patterning the vertebrate neural tube [21] (although it is not clear if the underlying mechanism is truly a speed regulation model or a dynamic version of the French Flag model; see [10] for a discussion).

#### The clock

**Fig 7:**
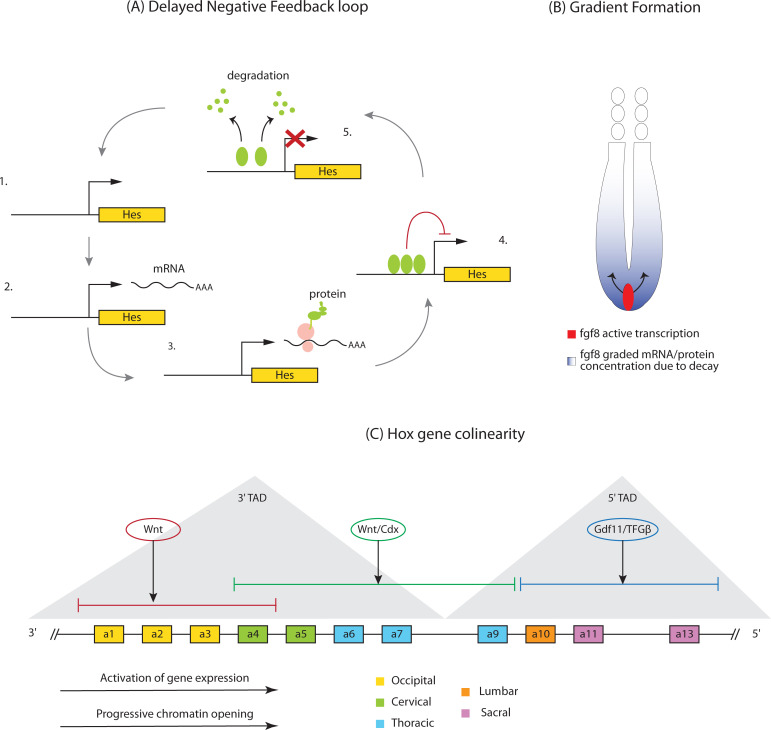
Mechanisms of segmentation and regionalization in vertebrates. **(A)** Delayed negative feedback loop giving rise to oscillations in Hes/Her expression. In the absence of transcriptional repression by autoinhibition (1), Hes/Her genes are activated and mRNA transcribed (2). This leads to HES/HER protein translation (3) and accumulation. After a time delay associated with gene expression steps, HES/HER proteins reach sufficient levels to bind the Hes/Her regulatory regions and inhibit transcription (4). Autoinhibition is relieved by HES/HER degradation (5) and the cycle begins again. **(B)** Mechanism of FGF gradient formation. Only progenitor cells (pink) actively express the *fgf8* ligand. Once cells ingress into the PSM (purple), they cease to transcribe *fgf8*. Progressive degradation of *fgf8* mRNA and protein leads to gradient formation as cells acquire more anterior positions within the paraxial mesoderm. **(C)** Genomic organization of the HoxA cluster. HoxA1-13 genes are arranged colinearly within the cluster in the 3′ to 5′ orientation. Chromatin opening and gene expression start at the 3′ end and proceed in the 3′ to 5′ direction. Genes are colored according to the vertebral identities they specify. The 2 TADs (3′ and 5′) are shown as gray triangles. Anterior Hox genes are activated by Wnt signaling (red), central Hox genes by Wnt/Cdx (green), and posterior Hox genes by Gdf11/TGFβ (blue). PSM, presomitic mesoderm; TAD, topologically associated domain.

Irrespective of the details of the clock and wavefront model, which are still actively debated, it is clear that a molecular oscillator is at work in the PSM of vertebrates and SAZ of short-germ insects. Such oscillations in cyclic gene expression are thought to be generated by negative feedback loops with delays [157] (Fig 7A). In vertebrates, for instance, bHLH *Hes/Her* transcriptional repressors can inhibit their own promoters [158]. Once induced, accumulation of HES/HER protein leads to transcriptional silencing until such time as the proteins are degraded and transcription can resume once more. Synchronization of individually oscillating cells in vertebrates is then mediated by cell–cell coupling through Delta–Notch interactions. Inhibiting Delta–Notch signaling disrupts traveling waves and leads to a salt and pepper expression pattern of *Hes/Her* genes in both mouse and zebrafish [159–161]. In some (but not all) arthropods, Notch signaling has been shown to be involved in segmentation [61,63,66–68].

In amniotes, oscillations of targets of the FGF and Wnt signaling pathways are thought to arise from similar time-delayed negative feedback loops. FGF and Wnt ligands provide constant pathway activation in the posterior PSM, resulting in the induction of target genes like *Dusp* or *Axin2*, respectively. As these genes are involved in feedback inhibition (i.e., dual-specificity phosphatase (DUSP) dephosphorylates mitogen-activated protein (MAP) kinases; AXIN2 is a component of the β-catenin destruction complex), their expression shuts down pathway activity, and, hence, their own transcription [75,161]. However, negative feedback loops cannot fully account for the mechanism of segmentation clock oscillations, as overexpressing constitutively active β-catenin does not impair Wnt or Notch oscillations in the mouse PSM [162]. Thus, oscillations might be generated by noncanonical regulation of Wnt and FGF targets. In mouse embryonic stem cells, desynchronized oscillations of many genes with the same period as the mouse segmentation clock have been observed [163]. This suggests that oscillatory gene expression dynamics might be more widespread than currently appreciated.

In *Tribolium*, 3 primary pair-rule genes (*eve*, *run*, and *odd*) are thought (arguably, [12,113]) to be wired into a genetic clock that produces their oscillatory expressions [60] (Fig 4C). This hypothesis is supported by RNA interference (RNAi) knockdown experiments: Knocking down *eve* results in the down-regulation of both *run* and *odd*, knocking down *run* leads to the down-regulation of *odd* and the overexpression of *eve*, and knocking down *odd* leads to the overexpression of both *eve* and *run* [60] (Fig 4D). These experiments suggest that *Tribolium eve*, *runt*, and *odd* are wired into a negative feedback loop in the same order as they appear in the SAZ (Fig 4C). Knocking down 1 or more of the primary pair-rule genes has been found to have a similar (but not identical) disruptive effect on the overall pair-rule pattern in other short-germ insects as well [31,32,164,165]. So far, there is no evidence that Notch signaling is involved in segmentation in *Tribolium* [67,68].

#### The wavefront

The segmentation clock must interact with the determination front (or wavefront) to establish segment boundaries (Box 3). In vertebrates, this determination front is positioned by gradients of FGF, Wnt, and RA signaling [166]. Experimentally manipulating these gradients artificially displaces the wavefront. For instance, transient FGF inhibition shifts the wavefront posteriorly and results in larger somites [91,167]. These signaling gradients are formed by a combination of localized ligand production, progressive ligand decay, and ligand diffusion. In the case of FGF signaling, *Fgf8* mRNA is actively expressed at high levels in the posterior progenitor zone [92]. However, once cells are specified as mesodermal and ingress into the PSM, they cease to express this gene (Fig 7B). As the embryo continues to elongate posteriorly and cells progressively acquire more anterior positions, dilution and degradation of existing *Fgf8* mRNA molecules give rise to the posterior–anterior gradient. FGF8 protein decay has not been directly measured, but also certainly plays a role in establishing the gradient. In addition to this decay mechanism, FGF ligand diffusion takes place in the PSM and contributes to gradient formation [94]. A similar mechanism probably underlies the Wnt3a gradient, as this signaling molecule is only transcribed in the tail bud and posterior PSM [73]. Given that Cdx genes are targets of both Wnt and FGF signaling in vertebrates, a parallel posterior-to-anterior Cdx gradient is formed downstream of these pathways [168–170]. Progressive Cdx mRNA and protein degradation also contributes to Cdx gradient formation [171]. In the case of the anterior-to-posterior RA gradient, the RA-synthesizing enzyme *Raldh2* is expressed in the somites and anterior PSM, whereas the RA-degrading enzyme *Cyp26A1* is expressed in the tail bud [172,173]. This gradient is thus formed by a source-and-sink mechanism coupled with high RA diffusivity.

In *Tribolium*, the wavefront is thought to be encoded by Wnt and/or Cad gradients, since their expressions overlap pair-rule oscillations in the SAZ [8,13] (see *cad* expression in Fig 2B; compare with pair-rule gene expressions in Fig 2A). Once out of the domain of Wnt/Cad expression, pair-rule oscillations are fixed into stripes. The expressions of *opa* and *dichaete* were recently shown to form staggered wavefronts along with Wnt/Cad [13]. Whereas *opa* was suggested to mediate the frequency doubling of pair-rule stripes upon their exit from the SAZ (see below), it is not clear if *opa* and/or *dichaete* are involved in slowing down and arresting pair-rule waves intro stripes, nor how they interact with the Wnt/Cad gradient.

Posterior Wnt activity in *Tribolium* is mediated by both *wnt1* and *wnt8* ligands, which are expressed posteriorly in the embryo [85–87]. Wnt then activates *cad* resulting in a parallel Cad gradient that itself activates wnt1, forming a positive feedback loop [8,85]. In the *Tribolium* blastoderm, the Wnt/Cad gradient is first established by a maternal counter gradient of the Wnt negative regulator *axn* and is additionally modified by the repressing effect of *zen* and *otd* counter gradients [8,85]. Wnt/Cad gradient formation and retraction during the germ-band stage is not well studied, but in principle could be mediated through RNA/protein decay, similar to FGF gradient formation in the vertebrate PSM.

#### Segment specification and polarity

In vertebrates, the determination front set up by Wnt and FGF signaling pathways functions to endow cells with the competence to respond to the clock signal and establish the future somite prepattern. Upon stimulation by the segmentation clock, cells in the determination front transiently stabilize Notch signaling in the form of a bilaterally symmetric stripe of Notch activation that can be visualized by Notch intracellular domain (NICD) expression [186]. Notch activity results in the TBX6-dependent expression of *Mesp1/2*, the master regulators of the segmental program [187]. Following segment specification, *Mesp1/2* become restricted to the rostral part of the future somite, whereas Delta-like1 (*Dll1*) and NICD are restricted to the caudal part [77,188]. This results in the establishment of somite rostral–caudal polarity. *Mesp1/2* expression is eventually down-regulated through a negative feedback loop involving its target *Ripply2* and *Tbx6* [189,190]. Somite polarity is subsequently maintained by the transcription factors *Tbx18* (rostral) and *Uncx4*.*1* (caudal), which functionally antagonize each other [191].

In *Tribolium*, as the expression waves of the primary pair-rule genes *eve*, *run*, and *odd* propagate out of the SAZ, they slow down and eventually refine their expression patterns and undergo frequency doubling [3,6,13,60]. In addition, primary pair-rule genes regulate the secondary pair-rule genes paired (*prd*) and sloppy-paired (*slp*) at the border of the SAZ [60]. The later phase of pair-rule frequency doubling has been suggested to be mediated by *opa*, which is expressed along with Wnt/Cad and *dichaete* in a staggered wavefront [13].

The refined expressions of the *Tribolium* primary and secondary pair-rule genes constitute a combinatorial code that divides each clock-mediated pair-rule wave into 2 parasegments and defines their polarities. The anterior of odd-numbered parasegments is demarcated by the expression of segmental *eve* stripes and *prd*, whereas the anterior of even-numbered parasegments is demarcated by the expressions of *eve*, *run*, *odd*, and *prd*. The posterior of all parasegments is demarcated by *slp* expression. This combinatorial code eventually regulates the segment polarity genes *en* and *wg* at the anterior and posterior of each parasegment, respectively [80], in a fashion similar to segment polarity gene regulation in *Drosophila* [192].

#### How do clock oscillations organize into traveling waves across vertebrate and short-germ insect embryos?

When *cHairy1* oscillations were first discovered in the chick PSM, they were shown to be expressed in periodic waves that sweep the PSM from posterior to anterior, progressively slowing down and becoming narrower as they approach the determination front [4,193,194], an observation not accounted for in the original formulation of the clock and wavefront model. These waves do not require diffusion or cell–cell communication, and, hence, are called “kinematic” or “pseudo-waves” [4,195] (although the involvement of cell–cell communication might still be necessary to ensure synchronous oscillations in neighboring cells [159]). A simple model for how such waves are produced is to assume that the regressing wavefront modulates the frequency of the segmentation clock in a dose-dependent fashion, acting, consequently, as a regressing frequency gradient (Box 3) [4,179]. The mechanisms underlying this frequency gradient are currently not well understood. Although there is some evidence suggesting that traveling waves are initiated and controlled by FGF through a posterior-to-anterior phase delay [196], traveling waves can still take place in the absence of an FGF gradient [197]. Interestingly, different cyclic genes display distinct frequency profiles. For instance, in mice, the Wnt target *Axin2* displays rapid waves that slow down abruptly, whereas waves of the Notch target *Lfng* travel more slowly [76].

The functional importance of the gradual slowing down of clock oscillations is unclear, since, theoretically, the performance of the clock and wavefront model is basically the same whether oscillations are arrested suddenly in a catastrophic event (as in the original formulation of the model) or through a gradual slowdown [8,179]. However, during the blastoderm stage, *Tribolium* pair-rule genes were shown to be expressed in waves in the absence of axis elongation or a regressing wavefront (Fig 2A), a fact that cannot be explained by the original formulation of the clock and wavefront, but rather by the action of a nonregressing frequency gradient [6]. This highlights the importance of the gradual slowdown of clock oscillations in certain scenarios. Oscillations of the segmentation clock, therefore, can be translated into periodic spatial patterns either by a regressing wavefront or by a nonregressing frequency gradient, a fact that is elucidated in a unified model for time-based patterning called the “speed regulation” model (Box 3) [3,174]. The model describes a core regulatory mechanism in which a gradient of a molecular factor (called the speed regulator) regulates the speed of the segmentation clock (or any temporal process). A nonregressing gradient of this speed regulator can by itself induce oscillatory waves, and, hence, pattern nonelongating tissues like insect blastoderms (Fig 6B, left bottom panel; Box 3), whereas a regressing gradient of the speed regulator can pattern elongating tissues like insect germ-bands and vertebrate PSMs (Fig 6B, bottom right panel; Box 3). Interestingly, the formation of the prechordal and head mesoderm in vertebrates is associated with 2 cycles of oscillatory gene expression that take place in the epiblast without a regressing wavefront and might be conceptually similar to pair-rule pulses in the *Tribolium* blastoderm [198].

Hence, a single model can explain both blastoderm and germ-band segmentation in short-germ insects [3]. The Wnt/Cad gradient has been suggested to act as a speed regulator for the pair-rule oscillator in *Tribolium* [3,8]. Experimentally manipulating the Wnt/Cad gradient in several genetic backgrounds led to stereotypical changes in the spatiotemporal dynamics of pair-rule waves [8] (Fig 4G). In most cases, reducing the intensity of the Wnt/Cad gradient led to slower pair-rule wave dynamics, shifting the Wnt/Cad gradient anteriorly or posteriorly led to a concomitant anterior or posterior shift of pair-rule waves, respectively, and stretching the Wnt/Cad gradient led to a stretch in pair-rule waves (Fig 4G). Using computational modeling, these observations were shown to be consistent with the speed regulation model [3,8]. However, these conclusions are based on correlations between the Wnt/Cad gradient and pair-rule wave dynamics, and it is still unclear whether other factors are also involved (like *opa* and *dichaete* [13]) or what molecular mechanisms underlie such speed modulation of segmentation clock oscillations (more on that below). Most of these observations are made during the early phase of segmentation in the *Tribolium* blastoderm, where other maternal and zygotic gradients exists (e.g., *otd* and *zen*) that could be directly involved in regulating pair-rule gene regulation. Moreover, it is unclear if the same model is applicable to later stages of segmentation in the germ-band. However, in *axn* RNAi embryos, the *cad* gradient was shown not to regress in the *Tribolium* germ-band. Concomitantly, gap gene waves (discussed in section “Mechanisms of regionalization in short-germ insects”), which are nonperiodic version of pair-rule waves, were observed to continue propagating and shrinking without being arrested into stable expression domains in this genetic background, as predicted by speed regulation model [3]. This suggests that regionalization, and potentially segmentation, rely on the same core mechanism in both the blastoderm and the germ-band.

In vertebrates, it has recently been reported that pharmacological inhibition of FGF leads to a dose-dependent lengthening of the oscillatory period for the *Lfng* reporter *LuVeLu* in mouse PSM explants [184]. Furthermore, treatment of mouse and chicken PSM with Wnt inhibitors can alter the frequency profile of traveling waves [199]. These studies suggest that gradients of FGF and Wnt signaling might act as speed/frequency regulators in vertebrate embryos similar to how Wnt/Cad gradients have been suggested to act in short-germ insects [8].

In line with the role of Wnt/FGF signaling in frequency modulation in amniotes, sustained oscillations were observed in mouse PSM explants cultured under experimental conditions that maintained a uniform level of Wnt and FGF activities, without any sign of variability in frequency [197]. On the other hand, explants under normal conditions, in which Wnt and FGF gradient are established, a gradient of oscillation frequencies is observed [185]. Interestingly, however, in the absence of Wnt and FGF gradients, oscillatory waves are still observed in mouse PSM explants [197]. These waves, however, show no sign of progressive reduction in length as seen in explants cultured under normal conditions. This indicates that in the absence of a frequency gradient (presumably mediated by Wnt/FGF signaling gradient), cells still self-organize into spatial waves, potentially through the emergence of phase differences between neighboring cells.

But if the frequency of the segmentation clock is modulated along the AP axis via a speed regulator gradient, how could this be achieved at the molecular level? In a recently devised model of AP patterning in *Tribolium* [3,200], segmentation genes where proposed to be wired into 2 different gene regulatory networks: a clock network and a multistable gene network (Fig 4I). If a Wnt/Cad speed regulator activated the clock network but repressed the multistable network, a gradual slowdown in oscillation will result along the speed regulator gradient (Fig 4I). A similar model was suggested for gap gene regulation in *Tribolium* (replacing the clock with a genetic cascade). In line with this model, reactivating the gap gene *hb* all over the embryo resulted in 2 distinct responses [201]. Within the Wnt/Cad expression (defining the SAZ), the gap genetic cascade was reset, and the whole gap gene sequence was reproduced. Anterior to the SAZ, the already formed gap genes expressions were erased [10] (Fig 4H). The 2 different responses of the gap gene network to the same perturbation within and outside of the SAZ indicates that gap genes are regulated by 2 different genetic programs: one within the SAZ and one outside and were suggested to be mediated by 2 different groups of enhancers active at the 2 regions [3,10]. This is in line with the observation that the gap gene *Kr* in *Drosophila* is regulated by 2 enhancers, one initiates its expression and the other maintains it [52]. Interestingly, open chromatin at early acting enhancers in the *Drosophila* embryo has been shown to be mediated by the pioneer factor Zelda and at late-acting enhancers by the pioneer factor Opa [143,144]. One could imagine then a scenario in which a gradual switch between early and late-acting enhancers in *Tribolium* to be mediate by opposing gradients of *opa* [13] and *zelda* [145], where the expressions of *opa* and/or *zelda* are regulated by (or interact with) the Wnt/Cad gradient. Interestingly, in vertebrates, it was shown that some enhancers and/or genetic programs mediate the initiation of segmentation clock waves posteriorly, and others mediate their anterior expressions [202–204].

In an alternative hypothesis, the period of the segmentation clock could be spatially modulated by a protein production time delay that increases along AP axis [205]. In yet another hypothesis, the period of the segmentation clock is regulated by the intercellular coupling delay. In vertebrates, individual PSM cells form a system of phase-coupled oscillators whose synchronization is mediated by Delta–Notch signaling [159–161]. The strength of this coupling has been proposed to modulate the period of collective oscillations [206,207], and, therefore, a gradient of coupling strength could mediate the observed frequency gradient. In zebrafish, disrupting Notch signaling either genetically or pharmacologically leads to a moderate increase in somitogenesis and clock period [207]. In *Lfng* mutant mice, the coupling delay is shortened, and oscillatory period is concomitantly decreased in intact PSM tissue but not isolated PSM cells [206]. Consistently, primary mouse PSM cells and in vitro–derived human PSM cells both maintain their oscillatory period when cultured at low densities such that no cell–cell signaling can take place [184,206]. The HES7 oscillatory period is unchanged even when isolated cells are treated with Notch inhibitors or obtained from *Lfng* mutant mice [184,206]. These results indicate that the segmentation clock pacemaker acts in a cell autonomous way, but the collective period can be modulated in the tissue context through cell–cell coupling.

#### Inter- and intraspecies plasticity of segmentation clock frequency

Clock-based segmentation programs enable the conversion of periodic temporal patterns (oscillations) into spatial patterns (segments). However, the pacemaker mechanisms that control the periodicity of the clock itself remain poorly understood. Strikingly, the oscillatory period of cyclic genes displays great plasticity in both vertebrates and arthropods. First, traveling waves slow down and become narrower as they reach the anterior part of the PSM or SAZ as described above. Second, the somitogenesis period in vertebrates is not constant throughout axis formation but rather increases gradually as the caudal most somites are laid down [208]. An extreme example is the marsupial *Monodelphis domestica*, which displays a lengthening in somitogenesis period from 1 hour at cervical levels to 4.5 hours at caudal levels [209]. Similar observations have been made in *Tribolium*, where the rate of segment addition varies along the AP axis [57,210].

Like the slowdown of traveling waves along the PSM/SAZ, and in line with the speed regulation model (Box 3), the gradual lengthening of clock period toward the end of axis elongation may potentially be explained by changes in the level of speed regulators with time. Axis termination in vertebrates involves the down-regulation of Wnt and FGF signaling, which may account for the longer somitogenesis period at these stages [117].

In addition to clock frequency variation along the AP axis, the period of the segmentation clock varies significantly across species. The segmentation clock oscillates every 30 minutes in zebrafish [208], 1.5 hours in chicken [4], 2.5 hours in mouse [211], 3 hours in *Tribolium* [6], and 5 hours in human [72,184]. This parameter is strongly correlated with developmental speed, such that more rapidly developing species like zebrafish exhibit shorter clock periods. Furthermore, the somitogenesis period, like developmental rate, is temperature sensitive. Raising embryos at different temperatures leads to scaling of the clock period in both vertebrates and arthropods [6,208,212,213]. This tight coupling between segmentation clock period and developmental speed has resulted in the emergence of somitogenesis as a model for the study of allochrony [214].

Currently, the predominant hypothesis concerning the segmentation clock pacemaker in vertebrates is based upon the *Hes/Her* time-delayed negative feedback loops that are thought to underlie cyclic gene oscillations [158]. The cumulative time of gene expression steps such as transcription, splicing, nuclear export, and translation of *Hes/Her* genes are referred to as the transcriptional delay and represent the total time delay before feedback inhibition can take place [157]. These transcriptional delays have been proposed to regulate the oscillatory period across species. Indeed, transcriptional delays for *Hes/Her* orthologs are longer in vertebrate species with slower clock period, and removing introns from the mouse *Hes7* gene to accelerate splicing results in a moderate acceleration of oscillations [215,216]. These interspecific differences in *Hes/Her* gene expression kinetics are most likely the result of larger differences in the global rates of biochemical reaction speeds between species. Recent studies have shown that the production and degradation of many proteins, not only Hes/Her proteins, is accelerated in mouse PSM cells compared to human cells [214]. Similarly, the global half-life of the mouse proteome is significantly shorter in mouse neural progenitors than human [217]. It thus seems that species-specific segmentation clock periods are the result of global scaling in gene expression and protein turnover rates. Further work is required to understand the mechanisms that underlie and regulate species-specific biochemical reaction speeds.

These studies also suggest that Hes/Her genes may not represent the core segmentation clock pacemaker, at least in mouse. Rather, the clock period may be an emergent property of system-wide kinetics. In line with this hypothesis, mice carrying null alleles of *Hes7* still exhibit residual segmentation, and the period of Wnt oscillations remains unchanged [218,219]. Mouse embryonic stem cells where the entire *Hes7* locus has been swapped for its human ortholog differentiate into PSM cells that oscillate with the characteristic mouse-specific period, thus confirming that differences in Hes7 genomic sequence cannot explain the interspecies differences in clock period [214]. *Hes7* is thus unlikely to be the fundamental pacemaker for the mammalian segmentation clock. Alternatively, other Hes genes may work redundantly with Hes7 as pacemakers, thus complicating the interpretation of the single mutant phenotype.

### Mechanisms of regionalization in short-germ insects

As discussed earlier, the predominant model of gap gene regulation in the long-germ insect *Drosophila* is based on a French Flag mechanism, in which the maternal Hb gradient acts as a master regulator. The maternal Hb gradient, however, plays less prominent role in *Tribolium*: Flattening the maternal Hb gradient in *Tribolium* (by knocking down *nos*;*pum*) only slows down the onset of the sequential activation of gap genes [9]. Alternatively, the posterior-to-anterior gradient of Wnt/Cad was proposed to be a master regulator of gap genes in *Tribolium* [3,201]. Depleting *cad* by RNAi completely abolishes gap gene expressions in *Tribolium*, and manipulating the Wnt/Cad gradient in several genetic backgrounds leads to stereotypical changes in the spatiotemporal dynamics of gap gene expression waves similar to those observed for pair-rule genes in the same genetic backgrounds [3,8]. These observations led to the proposal that gap genes, similar to pair-rule genes, are regulated according to the speed regulation model, in which Wnt/Cad acts as a speed regulator (Box 3) [3]. In this model, gap genes are wired into a genetic cascade that mediates the observed sequential activation of gap genes (Fig 4E). The wiring of gap genes into a genetic cascade is supported by RNAi knockdown experiments (Fig 4F): Knocking down one gap gene leads to the down-regulation of downstream gap genes and the up-regulation of upstream gap genes [110–113,220] (similar gap gene phenotypes were also reported in the milkweed bug [221]). The model then presumes that the Wnt/Cad gradient acts to modulate the speed of the genetic cascade, and, hence, the speed of the sequential activation of gap genes. During the blastoderm stage, the *cad* gradient is nonregressing and induces sequential waves of gap genes according the nonperiodic and gradient-based version of the speed regulation model (Box 3; Fig 6A, bottom left). During the germ-band stage, the *cad* gradient starts to regress, producing more gap gene waves, where gene expression patterns left behind by the *cad* gradient are arrested into stable gene expression domains [3], according to the nonperiodic and wavefront-based version of the speed regulation model (Box 3; Fig 6A, bottom right).

Gap gene expressions in *Tribolium* are the main regulator of downstream Hox genes. Little experiments, however, have been done to study Hox gene regulation in *Tribolium* other than those showing their disruption upon gap gene knockdowns (summarized in [33,113]), and it is unclear if Hox genes are involved in axis termination in this insect as the case in vertebrates.

### Mechanisms of regionalization in vertebrates

In most vertebrates, genome duplications have resulted in 4 separate Hox clusters (*HoxA-D*) [222]. Mammalian genomes thus encode 39 individual Hox genes organized into 4 clusters and 13 paralogous groups [222]. There is extensive functional redundancy between the paralogs, so much so that deleting entire Hox clusters does not impair segmental identity [223,224]. Despite the duplication of Hox gene clusters, colinear expression within each cluster has been maintained. The regionalization of segments by Hox genes has been most intensively studied with regard to patterning of the vertebral column in the axial skeleton. The spine is divided into morphologically and functionally distinct regions corresponding to the cervical, thoracic, lumbar, sacral, and caudal vertebrae. While the specific axial formula varies between species, the role of Hox genes in specifying distinct regional identities appears to be conserved (Fig 7C). Specifically, *Hox4* genes control patterning of the cervical somites that form the neck [225], whereas the *Hox5*, *Hox6*, and *Hox9* paralogous groups are involved in the development of thoracic segments [226]. Moreover, *Hox10* paralogs are required for the specification of lumbar identity and are capable of suppressing rib formation when overexpressed in the thoracic region [227]. Similarly, *Hox11* genes are essential for sacrum specification and *Hox13* genes correspond to the caudal vertebrae [227,228].

The Hox gene expression domains that determine axial identity are thought to be established by a “Hox clock” that drives the sequential activation of Hox genes in the progenitor domain [11]. This sequential activation follows the order of the arrangement of Hox genes within each cluster and is thus temporally colinear (Fig 7C) [106,229]. Hox expression in the progenitor domain controls the timing of mesodermal cell ingression, such that progenitors expressing only anterior Hox genes ingress earlier and end up in more anterior positions along the body axis [116,117]. Posterior Hox genes (paralogs 9 to 13) delay cell ingression in a graded fashion, with *Hox13* paralogs displaying the strongest effect and also triggering axis termination [117]. Thus, temporally colinear Hox expression coupled with timed cell ingression in the progenitor domain set up the nested spatial colinearity that confers axial identity. Once progenitor cells ingress, they maintain their Hox gene expression, and in this way, the sequential activation of Hox genes is translated into stable spatial domains [116,230]. This mechanism is similar to the clock and wavefront, where cell ingression acts as a determination front that translates the ticks of the Hox clock into spatial pattern (or, equivalently, similar to the nonperiodic wavefront-based mode of the speed regulation model; see Box 3 and Fig 6A, bottom right panel).

#### The Hox clock

What drives the sequential activation of Hox genes is highly debated. Several lines of evidence argue for a functional relevance of the fact that the 3′ to 5′ organization of the Hox cluster matches the temporal sequence of Hox gene activation. All genes in a Hox cluster are initially in a compact chromatin state that prevents transcription [231]. Opening the chromatin then proceeds sequentially beginning at the 3′ end [229]. The progressive opening of the chromatin is mediated by the clearing of repressive histone modifications such as H3K27 trimethylation, which are laid down and maintained by the Polycomb repressive complex [231]. These inactivating histone marks are, in turn, replaced by activating ones, including H3K4 trimethylation. The “open for business” model suggests that there might be an intrinsic mechanism that mediates this progressive opening of chromatin with time, and consequently, the sequential activation of Hox genes [11,232]. Indeed, the order of Hox genes within a Hox cluster is important. Flipping the orientation of a HoxD cluster such that HoxD13 gene becomes located 3′ in the cluster leads to embryonic lethality [233]. Furthermore, chromatin architecture is crucial to the correct sequential activation of Hox genes, as insulation through CTCF establishes topological boundaries that prevent premature expansion of the active domain [234]. In addition, mouse Hox clusters were found to be in between 2 separate topologically associated domains (TADs) that have been proposed to be important for their colinear activation [235].

However, it is so far unclear whether chromatin regulation is the main driver of Hox colinearity or just a safeguard to prevent the accidental premature activation of more posterior Hox genes, which exhibit dominant negative effects over anterior ones. It has been recently shown that a relay mechanism (or a genetic cascade) composed of cross-regulatory interactions between Wnt, Cdx, and Gdf11/TGFβ acts upstream of Hox genes and (partly) mediates their collnear activation [229,236–238], reminiscent of the genetic cascade that drives the sequential activation of gap genes in *Tribolium*. Early Hox genes (which are anteriorly expressed and located at 3′ end of the cluster) are activated by Wnt signaling through asymmetric distribution of Wnt-responsive enhancer sequences on the 3′ side of the cluster [229]. Later on, Wnt activates Cdx, which, in turn, activates more centrally located Hox genes through binding sites within the Hox cluster [236]. Finally, more 5′-located Hox genes are activated by Gdf11/TGFβ signal in the tail bud [237,239]. Hence, a sequence of Wnt, Cdx, and TGFβ signals mediates the sequential activation of the 3′, central and 5′ subdomains of the Hox cluster, respectively (Fig 7C). The sequential activation of Hox genes within each subdomain is so far not well understood and could rely on an intrinsic mechanism of progressive chromatin opening and/or a cross-regulatory scheme between Hox genes [240].

#### Hox gene waves

Hox genes of paralogous groups 1 to 8 are first expressed sequentially in a salt-and-pepper pattern in the posterior region of the primitive streak [116]. The expression domain for each Hox gene then expands anteriorly until it encompasses the entirety of the primitive streak and adjacent epiblast and part of the neural plate [230,241]. Anterior Hox genes are thus initially expressed in waves that sweep the epiblast from posterior to anterior (a phenomenon termed “spreading”), reminiscent of the nonperiodic waves of gap genes in short-germ insects. It is not known how these waves are induced, but could possibly be due to temporal modulation of Hox gene activation by early expressed Wnt and Cdx genes in the epiblast (see gradient-based mode of the speed regulation model, Box 3; Fig 6, left bottom panel) or alternatively through a French Flag model (Box 1). Interestingly, loss of Cdx results in posterior shifts and delay in timing of central Hox genes [242], reminiscent of experiment in which *cad* concentration is reduced in *Tribolium* (Fig 4G) [3,8]. Moreover, levels of Fgf and Gdf11 were recently found to modulate the speed of sequential Hox gene activation in differentiating human pluripotent stem cells [243].

#### Hox genes and axis termination

In addition to specifying tail identity, the *Hox13* paralogous group has been proposed to control axis termination. In mice, loss of *Hoxb13* leads to the formation of extranumerary caudal vertebrae [228], while its overexpression results in premature truncation of the tail [244]. Similarly, zebrafish embryos that inducibly overexpress Hoxa13b also display severe axis truncation [245]. Through a genetic cascade involving the factors *Gdf11* and *Lin28*, the paralogs *Hoxb13* and *Hoxc13* trigger a reduction in the posterior progenitor pool of the tail bud [239]. As this pool is progressively depleted, axis extension slows down and is eventually halted. The speed of somitogenesis, nevertheless, remains stable, such that the length of the PSM is reduced with every cycle of somite formation until all paraxial mesoderm is segmented. The mechanisms underlying this process involve the down-regulation of Wnt and FGF signaling in the tail bud by *Hox13* paralogous group genes [117]. At the same time, these terminal Hox genes also inhibit expression of the RA-degrading enzyme *Cyp26A1* in the chicken and fish tail bud, and, hence, up-regulate RA signaling [244,246]. Axis termination in vertebrates is thus mediated by a decrease in Wnt and FGF signaling coupled with an increase in RA signaling, both of which are triggered by *Hox13* genes [117]. A similar mechanism might mediate the termination of AP patterning in short-germ insects. Indeed, a reduction in *cad* expression is observed by the end of germ-band elongation in *Tribolium* via an unknown mechanism, which could be mediated in principle by posteriorly expressed gap genes. Indeed, perturbing the gap gene *hb* leads to supernumerary segment formation in both *Tribolium* [10] and the silk moth *Bombyx mori* [247], indicating an interaction between gap and pair-rule genes either directly or indirectly through regulating the Wnt/Cad gradient.

## Coupling of segmentation and regionalization

Segments and fates along the AP axis of insects and vertebrates have species-specific registry. Any misalignment between regionalization and segmentation leads to incorrect segmental fates (homeotic transformation) that potentially leads to severe consequences on the organism fitness. Hence, the processes of segmentation and regionalization are usually coupled. How this coupling is carried out is well understood in *Drosophila*, where gap genes provide instructive cues for both segmentation (by regulating pair-rule genes) and regionalization (by regulating Hox genes). Pair-rule genes also coregulate Hox genes, providing an additional mechanism for coupling segmentation and regionalization. In *Tribolium*, although gap genes and pair-rule gene regulation represent 2 separate processes running in parallel, limited coupling between segmentation and regionalization has been documented [112,113].

In vertebrates, segmentation and regionalization are linked together through their common coupling to axial elongation. As described above, axial elongation takes place in the posterior progenitor domain, which corresponds to the regressing primitive streak at early stages and to the tail bud later in development. This posterior progenitor domain is not only the source of PSM cells but is also responsible for setting up the Wnt/FGF gradients that control segmentation and the point of origin for segmentation clock oscillations. At the same time, Hox gene expression patterns are also set up in this posterior progenitor domain, where they control the timing of mesodermal cell ingression [117]. This means that the groups of cells segmented into somites in the anterior PSM had been already patterned to express their corresponding Hox code much earlier in development. It has been suggested that the final allocation of somite axial identity is directly coupled to the segmentation clock, as several Hox genes display oscillatory expression in the amniote PSM and shifting somite boundaries leads to repositioning of Hox boundaries [82,248]. Similarly, loss of *Hoxb6* leads to somite formation and segmentation clock defects in tail bud-stage mouse embryos [249]. In contrast, conflicting evidence from slowly segmenting zebrafish mutants, which retain proper axial identity [250], has challenged the notion of direct coupling between the segmental program and regionalization. It thus remains unclear to what extent the segmentation clock plays a role in positioning Hox boundaries during somite formation.

## Toward reconciliation of simultaneous and sequential models of patterning

Although the long-germ insect *Drosophila* and the short-germ insect *Tribolium* utilize the same set of genes to mediate segmentation and regionalization of their AP axis, neither the gene expression dynamics nor the underlying patterning mechanisms seem to be similar in the 2 species. Specifically, both gap and pair-rule genes arise as sequential waves in *Tribolium*, whereas they arise more or less simultaneously in *Drosophila* (Fig 2). However, upon closer inspection, we find interesting similarities between patterning events in both species that hold promise for a reconciliation and a possible evolutionary path between sequentially segmenting (short-germ) and simultaneously segmenting (long-germ) insects.

**Fig 8:**
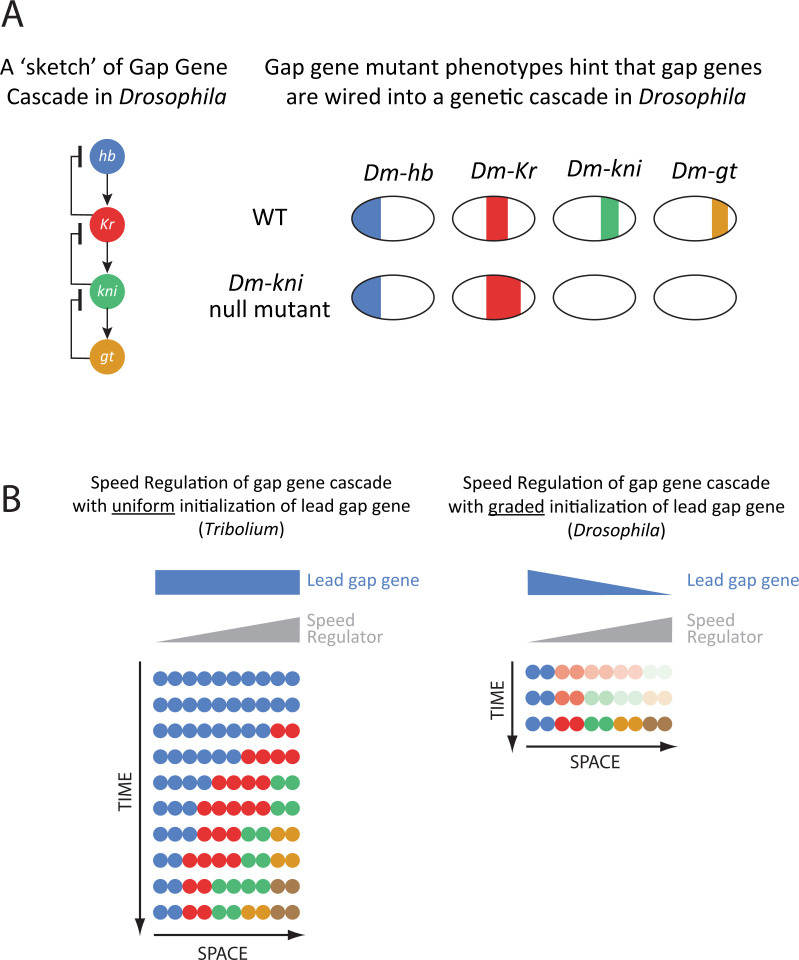
A model toward reconciliation of simultaneous and sequential modes of AP patterning in insects. **(A)** Right: In a loss of function mutant of a *Drosophila* gap gene (here shown only *Dm-kni* mutant), gap gene expression anterior to it is extended (here *Dm-Kr*), and gap gene expression posterior to it is missing (here shown for *Dm-gt*). This is reminiscent of the gap gene phenotypes in *Tribolium* (compare with Fig 4E and 4F) and suggests that gap genes in *Drosophila* are wired into a genetic cascade as well (see the sketch of a gene network to the left; note that this is a parsimonious wiring explaining observed gene expression dynamics in WT and mutant phenotypes, where wiring might differ from the one shown, especially that most gap genes are known to act as repressors rather than activators). **(B)** A model for the evolution of gap gene regulation in insects from a sequential mode of patterning (like in *Tribolium*) to a simultaneous mode (like in *Drosophila*). Left: Typical speed regulation model for producing nonperiodic patterns (Box 3), in which the leading gene in the gene sequence (blue) is initially uniformly expressed. Right: Expressing the leading gene in the gene sequence (blue) in a graded fashion result in speedy and seemingly simultaneous patterning [9]. AP, anterior–posterior.

Although gap gene expressions have been reported to arise more or less simultaneously in the blastoderm of *Drosophila*, recent studies using carefully staged in situ staining and live imaging demonstrated that gap gene domains, although arising de novo early on, undergo stereotypical posterior to anterior shifts [50,52] (Fig 2C), reminiscent of the wave dynamics of gap gene expressions in *Tribolium* (Fig 2B). This might be viewed as a manifestation of sequentiality in the gap gene activation in *Drosophila*, since a shifting border between 2 expression domains entails that cells at this border switch from expressing one gene to another [9,51]. This observation indicates that gap genes in *Drosophila* might after all be wired into a genetic cascade [9] (or a nonperiodic “oscillator” [51]) that mediates this limited form of sequentiality. Interestingly, gap gene mutant phenotypes in *Drosophila* further support this hypothesis: The loss of one gap gene domain (in a mutant of that gap gene) leads to the down-regulation of the gap gene domain immediately posterior to it and overexpression of the gap gene domain immediately anterior to it (Fig 8A) [9], reminiscent of the gap gene knockdown phenotypes in *Tribolium* (Fig 4F) [251–255] (see summaries of *Drosophila* gap gene mutant phenotypes in [9,34]). Based on these observations, a model was recently suggested to reconcile gap gene regulation in *Tribolium* and *Drosophila* [9,51]. In this model, the gap genes in *Drosophila*, like in *Tribolium*, are wired into a genetic cascade (Fig 8A). However, rather than regulating the speed of this genetic cascade via the posterior-to-anterior gradient of Cad (like in *Tribolium*; Fig 8B, left), the maternal Hb gradient *pre-sets* the *Drosophila* gap gene cascade at different initial phases along the AP axis to mediate simultaneous and fast patterning (Fig 8B, right) [3,9,51]. The same mechanism of gap gene initialization by a maternal Hb gradient was observed, to a lesser extent, during the blastoderm phase of *Tribolium* development [9].

Pair-rule gene regulation in *Tribolium* and *Drosophila* seem to be harder to reconcile. In *Drosophila*, pair-rule gene regulation heavily depends on instructive cues from upstream gap genes, whose expression patterns form significantly earlier than those of pair-rule genes. On the other hand, gap and pair-rule genes are expressed in parallel in *Tribolium* with limited interaction between the 2 gene classes, although the nature and extent of this interaction is currently unclear. A possible bridge between segmentation in the 2 species is the zebra enhancers of pair-rule genes in *Drosophila*. As discussed earlier, while each one or pair of pair-rule stripes in *Drosophila* is initially mediated by a stripe-specific enhancer, there exist late-acting “zebra enhancers” that drive the full 7-striped pattern of each pair-rule gene. While zebra enhancers were hypothesized to mediate the stabilization of the pattern already formed by the stripe-specific enhancers, they could have evolutionarily originated as a “clock enhancer” to mediate pair-rule oscillations in more ancestral sequentially segmenting insects (like *Tribolium*) [12]. The stripe-specific enhancers in these ancestral insects (and possibly *Tribolium*) could act later on to stabilize clock-oscillations into stripes (in a fashion similar to the 2-network model of gap gene regulation; Fig 4I), and possibly coupling their positions to that of gap gene expression domains. Another possibility is that in sequentially segmenting insects like *Tribolium*, the periodic expression of pair-rule genes is not generated by a dedicated clock at all, but by instructive cues from gap gene expressions. However, the complete breakdown of the periodic pair-rule expression upon knocking down any of the primary pair-rule genes in *Tribolium* [60], and the limited effect that gap gene knockdowns have on pair-rule expressions disfavors this model. A combination of a clock-based model and a model based on an instructive role by gap genes is still a possibility. In yet another model, ancestral insects like *Tribolium* might lack a full set of stripe-specific enhancers (although one study reports the existence of such elements for the pair-rule gene hairy in *Tribolium* [256]), and in the path to the evolution to a *Drosophila*-like mode of segmentation, stripe-specific enhancers regulated by gap genes might have then been added one by one, initially to ensure the robustness of segmentation, but later in evolution hijacked the process to mediate a fast and simultaneous mode of segmentation as observed in *Drosophila* [12].

## Conclusions and prospects

**Fig 9:**
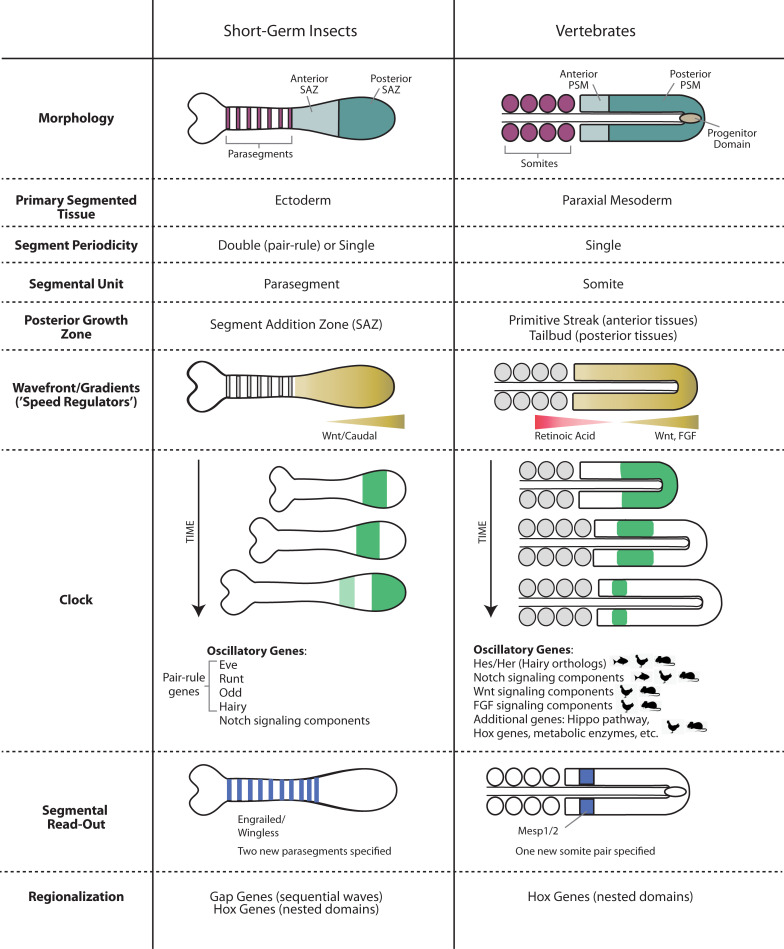
Comparison of segmentation and regionalization in short-germ insects versus vertebrates. PSM, presomitic mesoderm; SAZ, segment addition zone.

As highlighted in this review, vertebrates and short-germ insects share an impressive number of features related to segmentation and regionalization mechanisms (see a summary comparison in Fig 9). Patterning takes place as the axis elongates at the posterior end. The periodicity of segments is established by a genetic oscillator, whose frequency progressively decreases from posterior to anterior, generating traveling waves of gene expression. A determination front set by long-range signaling gradients freezes this periodic pattern into segmental units. Regionalization genes are activated sequentially under the influence of posteriorly localized gradients and are translated into nonperiodic expression patterns that divide the AP axis into different fates. These features are strikingly similar to patterning events in other tissues and organisms, suggesting that patterning with clocks and genetic cascades might be a widespread mechanism in development (Box 3).

Differences in segmentation and regionalization between insects and vertebrates are few but meaningful, such as the absence of both double-segmental patterning and gap gene equivalents in vertebrates. It is not possible to conclude whether these similarities and differences are the result of deep homology or convergent evolution. Nevertheless, if the urbilaterian ancestor was indeed segmented, it potentially patterned its segments sequentially through *Hairy/*pair-rule gene oscillations that interacted with a regressing determination front positioned by Wnt.

There remain many open questions in the field of segmentation and regionalization in both insects and vertebrates. What is the molecular mechanism underlying the speed regulation of the segmentation clock and regionalization genetic cascade? How is the segmentation clock wired? The amniotes segmentation clock seems to be composed of several constituents (Hairy and components of the Wnt, FGF, and Notch signaling pathways). Are these constituents independent, coupled (as indeed shown for Wnt and Notch), or all part of the same clock? Is the insect pair-rule clock composed of multiple coupled clocks? Is the wiring of this clock evolutionary flexible? [28–31]. How is segmentation and regionalization coupled? How are all these processes regulated at the *cis*-regulatory level? Finally, how could the sequential mode of AP patterning found in ancestral short-germ insects have evolved into a simultaneous one like that found in long-germ insects (e.g., *Drosophila*)? Several models for short- to long-germ evolution have been suggested in this and other articles [9,12,51] that still, however, await rigorous experimental verification.

In vertebrates, answering these questions has been hindered in part by the complexities of culturing and manipulating somitogenesis stage embryos, especially in the case of mammalian species. As an alternative, in vitro models of the mammalian segmentation clock have now been established [72,184,257]. Both the oscillatory gene expression of the segmentation clock and the temporally colinear activation of Hox genes have been recapitulated in 2D PSM cultures and 3D gastruloids derived from mouse and human pluripotent stem cells [184,258]. Traveling waves of the segmentation clock accompanied by a determination front can be generated in 3D PSM organoids as well as gastruloids [72,257,259]. In the case of gastruloids, spatial colinearity in the form of nested Hox expression domains is also observed [258]. In parallel, the genetic toolkit for sequentially segmenting insects such as *Tribolium* continues to grow. A tissue culture assay, RNAi screen, and a growing number of live imaging and genomic editing techniques have been developed for *Tribolium* [7,260–267]. Recently, a framework for enhancer discovery in *Tribolium* has been implemented [268], promising for a deeper understanding of segmentation and regionalization at the *cis*-regulatory level. When combined with the wealth of *cis*-regulatory data already available for *Drosophila* segmentation, this opens an avenue for understanding the evolution of patterning mechanisms at the molecular level.

These novel systems and techniques hold great promise to enable the detailed dissection of mechanisms controlling segmentation and regionalization in vertebrates and insects, and more generally, embryonic patterning mechanisms that rely on the spatial regulation of oscillatory and sequential gene activities.
